# Clinical utility of p16/Ki67 dual‐stain cytology for detection of cervical intraepithelial neoplasia grade two or worse in women with a transformation zone type 3: A cross‐sectional study

**DOI:** 10.1111/1471-0528.17248

**Published:** 2022-06-22

**Authors:** Line Winther Gustafson, Mette Tranberg, Pia Nørgaard Christensen, Rikke Brøndum, Nicolas Wentzensen, Megan A. Clarke, Berit Andersen, Lone Kjeld Petersen, Pinar Bor, Anne Hammer

**Affiliations:** ^1^ University Research Clinic for Cancer Screening, Department of Public Health Programmes Randers Regional Hospital Randers Denmark; ^2^ Department of Clinical Medicine Aarhus University Aarhus Denmark; ^3^ Department of Obstetrics and Gynaecology Aarhus University Hospital Aarhus Denmark; ^4^ Department of Pathology Randers Regional Hospital Randers Denmark; ^5^ Clinical Genetics Branch, Division of Cancer Epidemiology and Genetics National Cancer Institute Rockville Maryland USA; ^6^ Department of Obstetrics and Gynaecology Odense University Hospital and Open Patient Data Explorative Network Odense Denmark; ^7^ Department of Clinical Research University of Southern Denmark Odense Denmark; ^8^ Department of Obstetrics and Gynaecology Randers Regional Hospital Randers Denmark; ^9^ Department of Obstetrics and Gynaecology NIDO Denmark, Gødstrup Hospital Herning Denmark

**Keywords:** cervical intraepithelial neoplasia, colposcopy, human papillomavirus, large‐loop excision of the transformation zone, liquid‐based cytology, p16/Ki67 dual‐stain cytology, postmenopausal, transformation zone type 3

## Abstract

**Objective:**

To evaluate the clinical utility of p16/Ki67 dual‐stain (DS) compared with cytology for detecting cervical intraepithelial lesion grade two or worse (CIN2+) in women with a transformation zone type 3 (TZ3).

**Design:**

Cross‐sectional study.

**Setting:**

Colposcopy clinics in Central Denmark Region.

**Population:**

Women aged 45 years or older referred for colposcopy because of an abnormal screening test.

**Methods:**

All women had a cervical sample collected for cytology and DS testing and underwent large‐loop excision of the transformation zone (LLETZ).

**Main outcome measure:**

Sensitivity, specificity and negative (NPV) and positive (PPV) predictive values of DS for CIN2+ detection were compared to those of cytology.

**Results:**

Of 166 women eligible, 93 (56.0%) were included in the final analysis. Median age was 68 years (interquartile range [IQR] 63.4–70.5 years). Most women were postmenopausal (95.7%) and referred based on a positive human papillomavirus screening test (86.0%). Fifty‐two women (55.9%) were DS‐positive, 29 (55.8%) of whom had CIN2+ detected. Twenty‐seven (29.0%) women had atypical squamous cells of undetermined significance or worse (ASC‐US+), and CIN2+ was detected in 21 women (77.8%). DS had a higher sensitivity (96.7% versus 70.0% *p* = 0.021) and NPV (97.6% versus 86.4%, *p* = 0.018) compared with cytology for CIN2+ detection. In contrast, the specificity (63.5% versus 90.5% *p* < 0.001) and PPV (55.8% versus 77.8%, *p* = 0.001) were lower for DS compared with cytology.

**Conclusions:**

Dual stain may be a valuable risk marker to guide clinical management of women with a TZ3. The superior NPV of DS suggests that a diagnostic excision may safely be avoided in DS‐negative women.

## Tweetable abstract

DS‐negative postmenopausal women with a TZ3 have a low risk of CIN2+ and may undergo follow up instead of LLETZ.

## 1 INTRODUCTION

Cervical cancer screening aims to detect and treat cervical precancers, thereby reducing cervical cancer incidence and mortality.^1^ Screening with cytology and/or high‐risk human papillomavirus (HPV) testing may identify women who are at increased risk of disease, whereas colposcopy and biopsies are important parts of the diagnostic workup.^2^ In postmenopausal women, colposcopy is challenging because of atrophy, retraction and limited visualisation of the transformation zone (TZ).^3^ This may challenge the collection of biopsies, potentially resulting in increased risk of missing cervical intraepithelial neoplasia (CIN). In a recent publication we describe how biopsies missed more than half (54.5%) of the CIN grade two or worse (CIN2+) cases detected in specimens from large‐loop excision of the transformation zone (LLETZ).^4^ Scandinavian and Australian guidelines suggest that a diagnostic LLETZ may be considered in women with an abnormal screening result when visualisation of the TZ is incomplete (i.e. TZ3).^5^, ^6^, ^7^ The main disadvantages of LLETZ are the increased risk of overtreatment and complications such as stenosis, which may compromise follow up. In our recent study,^4^ 61% of women aged 45 years or older with a TZ3 had no disease detected in the LLETZ specimen, suggesting a significant risk of overtreatment if LLETZ is to be performed in all screen‐positive women with a TZ3. Therefore, a biomarker for correct risk stratification is urgently needed because this would enable a discrimination of women with a TZ3 at increased risk of CIN2+ who need excisional treatment from those who can safely undergo follow up. One such marker could be p16/Ki67 dual‐stain cytology (DS), which has been shown to provide better risk stratification of HPV‐positive women compared with cytology alone when used in primary screening.^8^, ^9^, ^10^, ^11^ However, it remains unclear if DS can be used as a tool to guide clinical management of women with a TZ3 at colposcopy.

This study aimed to evaluate the clinical utility of DS versus cytology for CIN2+ detection in a referral population of women with a TZ3.

## 2 METHODS

### 2.1 Setting and study design

This cross‐sectional study was conducted from March 2019 through June 2021 at the Departments of Obstetrics and Gynaecology in Central Denmark Region in collaboration with the Department of Pathology, Randers Regional Hospital, Denmark.

Denmark has an organised cervical cancer screening programme, with screening, clinical follow up and treatment offered free of charge. Women aged 23–59 years are screened with liquid‐based cytology (hereafter ‘cytology’), whereas women aged 60–64 years undergo HPV‐based screening. In addition, from April 2019 women aged 65–69 were invited for one additional HPV‐based screening test as part of an intervention study in Central Denmark Region.^12^ Women with abnormal screening results were referred for colposcopy and managed clinically according to national guidelines (Table S1).^7^, ^13^, ^14^

### 2.2 Participants and samples

Samples used in the present study were collected as part of another clinical study.^4^ In brief, women were eligible for enrolment if they were aged 45 years or above, referred for colposcopy because of an abnormal screening result (Table S1), and had a TZ3 at colposcopy according to the 2011 International Federation of Cervical Pathology and Colposcopy nomenclature.^15^, ^16^ Women were excluded if they wanted to become pregnant, if excision was not technically possible (e.g. pain, narrow vagina, atrophy or pelvic organ prolapse), had previous history of excisional treatment or hysterectomy, received anti‐coagulant medical treatment, or if they underwent repeated colposcopy for cervical dysplasia. Before enrolment, women received written and oral information about the study, and all included women signed an informed consent form. Before colposcopy, a cytology sample was collected in SurePath (BD Diagnostics, Burlington, NC, USA) for morphological assessment, and HPV and DS testing. All women had multiple biopsies taken for the purpose of another study and underwent a diagnostic LLETZ immediately after colposcopy using local anaesthesia (Citanest Dental Octapressin, Dentsply).

Women answered questions on basic characteristics (e.g. smoking, number of lifetime sexual partners, parity). Further, information on previous screening history was retrieved from the National Danish Pathology Databank^17^ using the woman's personal identification number, a unique code assigned to all Danish residents at birth or upon immigration.^18^ The National Danish Pathology Databank holds data on all cytopathological and histopathological examinations at an individual level for all Danish residents since 1997, and for some examinations back to 1970.^17^

### 2.3 Cytology, HPV and p16/Ki67 dual stain

Cytology slides were reviewed by experienced cyto‐technicians using computer‐assisted microscopy (BD FocalPoint GS Imaging System) and categorised using the Bethesda 2014 grading system.^19^ Cytology results were deemed invalid if too few squamous cells were present (i.e. ≤5000 cells per slide). Cytology results were grouped as normal cytology and atypical squamous cells of undetermined significance (ASC‐US+); low‐grade squamous intraepithelial lesions (LSIL); atypical squamous cells, cannot rule out high‐grade squamous intraepithelial lesion (ASC‐H) or high‐grade squamous intraepithelial lesion (HSIL). HPV DNA testing was performed using the clinically validated Cobas 4800 HPV DNA test (Roche Diagnostic) according to the manufacturer's instructions. This assay enables individual detection of HPV 16 and HPV 18, and pooled detection of 12 other high‐risk HPV types (31, 33, 35, 39, 45, 51, 52, 56, 58, 59, 66 and 68).^20^ DS was performed using the residual SurePath cell pellet in all included women regardless of HPV result. Details of the dual staining have been described elsewhere.^21^ In brief, DS was performed using the commercial US Food and Drug Administration approved CINtec PLUS assay (Roche Diagnostics).^22^ Slides were stained using the automated BenchMark ULTRA immunostainer (VENTANA, Roche Diagnostics) according to the manufacturer's instructions. Each staining run had one external cytology‐positive control (HSIL), and cells stained with either p16 or Ki67 were used as internal positive controls.

Dual‐stain slides were reviewed independently by two cyto‐technicians who were blinded to all study data (i.e. HPV genotype, cytology and histology results) except for the age of the woman. Both cyto‐technicians received training in interpretation of DS slides according to the manufacturer's recommendations.^21^ In case of discrepancy between the cyto‐technicians' results, a consensus‐based decision was performed based on a revision in a multi‐headed microscope. Slides were considered positive if one or more DS‐positive cells were detected, without consideration of morphology and cellularity criteria. For each slide the number of DS‐positive cells was recorded. Slides were deemed negative if no DS‐positive cell(s) were detected and the Bethesda 2014 criterion of squamous cellularity (i.e. ≥5000 cells per slide) was fulfilled. Slides were considered invalid if the cellularity criteria were not fulfilled or if one or both proteins (i.e. p16 or Ki67) were not stained. In case of an invalid DS slide, a second slide preparation and staining from the residual cell pellet was performed.

Cytology, HPV testing and DS testing were performed at the Department of Pathology, Randers Regional Hospital, Denmark, which is responsible for analysing all cervical cytology samples in Central Denmark Region (approximately 90 000 samples annually).

Clinical management was not based on DS results.

Histopathological examination of cervical biopsies and LLETZ specimens was performed in routine laboratories at the Departments of Pathology, Randers and Viborg Regional Hospital and graded according to the CIN classification^23^ as follows; <CIN2 (normal and CIN1) and CIN2+ (CIN2, CIN3, unclassifiable CIN [i.e. the full height of the epithelium is not discernible], adenocarcinoma in situ and cancer). The histopathological result of the LLETZ specimen served as the reference standard.^4^

No patients or patient organisations were involved in the development, design or implementation of this study.

### 2.4 Statistical analysis

For analysis, only women with valid results on cytology, HPV and DS were included. CIN2+ in the LLETZ specimen was used as the primary outcome instead of CIN grade 3 or worse because CIN2 is the threshold for excisional treatment in older women in most countries, including Denmark and the UK.^14^ Age and body mass index were calculated and presented as median with interquartile ranges (IQR).

For cytology and DS, we calculated specificity, sensitivity, positive predictive value (PPV) and negative predictive value (NPV) for CIN2+ detection with corresponding 95% confidence intervals (CI). Specificity and sensitivity for cytology and DS, respectively, were compared using the McNemar's chi‐square test. To test the robustness of our findings we conducted a sensitivity analysis excluding women with unclassifiable CIN. Further, we have restricted the analyses of DS positivity by cytology and histology to women who have undergone HPV‐based screening.

Finally, we calculated sensitivity, specificity, PPV and NPV using different thresholds of DS‐positive cells (1, ≥2, ≥6 and >50) for CIN2+ detection and calculated the Youden's index (YI = sensitivity + specificity − 1). Cases with one DS‐positive cell served as reference for the comparison between different thresholds.

Data were entered and stored in REDCap.^24^, ^25^ All statistical analyses were conducted using STATA version 17 (StataCorp LP, College Station, TX, USA), and for comparison of PPV and NPV we used the method developed by Leisenring et al. using the DTComPair package in R.^26^ Values of *p* less than 0.05 were considered statistically significant.

## 3 RESULTS

### 3.1 Study population

**FIGURE 1:**
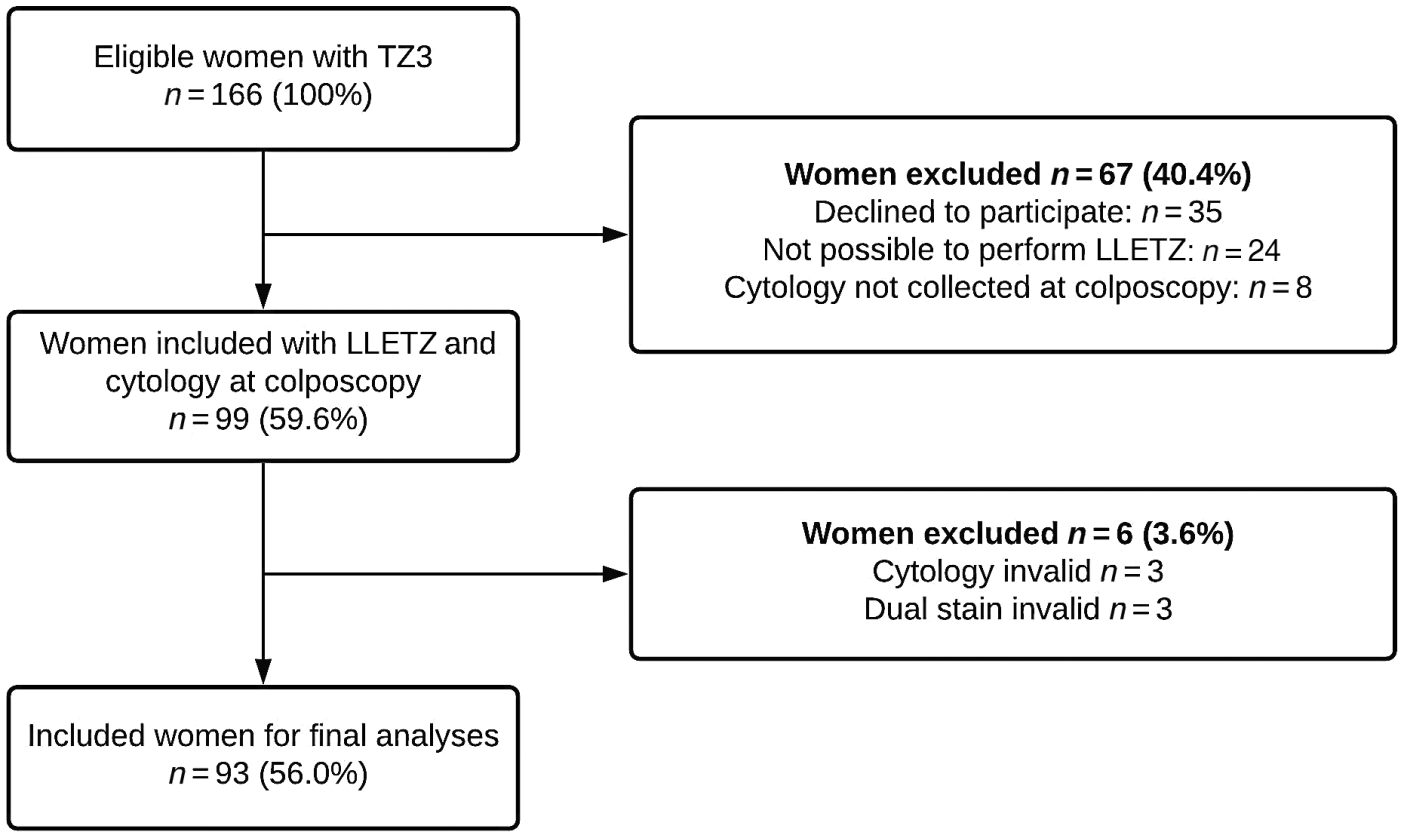
Flow chart for study population. LLETZ, large‐loop excision of the transformation zone; TZ3, transformation zone type 3

One‐hundred and sixty‐six women were assessed for eligibility, 99 (59.6%) of whom were enrolled (Figure 1). Six women (3.6%) were subsequently excluded because of invalid test results, leaving a total of 93 women (56.0%) for final analyses.

The median age of the included women was 68.0 years (IQR 63.4–70.5 years) with 89 (95.7%) women being postmenopausal (Table 1). Most women were non‐smokers (72.0%), parous (90.4%) and had no previous history of abnormal cytology (73.1%). The majority of women reported no new sexual partner within the past 2 years (83.9%), and 55 (59.1%) reported having five or more lifetime sexual partners. The majority had undergone HPV‐based screening with reflex cytology (82.8%) (Table 1).

**TABLE 1:** Basic characteristics for the included women (*n* = 93)

	Women
Age (years), median (IQR)	68.0 (63.4–70.5)
Body mass index (kg/m^2^), median (IQR)	24.4 (21.8–27.7)
Smoking, *n* (%)	
No	67 (72.0)
Yes	20 (21.5)
Missing	6 (6.5)
Menopausal status, *n* (%)	
Postmenopausal	89 (95.7)
Premenopausal	<3
Missing	<3
Parity, *n* (%)	
Nulliparous	5 (5.4)
Parous	85 (90.4)
Missing	3 (3.2)
Referral test, *n* (%)	
Primary HPV with reflex cytology	77 (82.8)
Primary cytology with reflex HPV test	4 (4.3)
Cytology only	9 (9.7)
HPV test only	3 (3.2)
Previous history of abnormal cytology (≥ASC‐US), *n* (%)	
No	68 (73.1)
Yes	25 (26.9)
Lifetime sexual partners, *n* (%)	
<5	27 (29.0)
5–10	36 (38.7)
>10	19 (20.45)
Missing	11 (11.8)
New sexual partners within the past 2 years, *n* (%)	
No	78 (83.9)
Yes	7 (7.5)
Missing	8 (8.6)
HPV vaccination, *n* (%)	
No	80 (86.0)
Yes	7 (7.5)
Missing	6 (6.5)

Abbreviations: ≥ASC‐US, atypical squamous cells of undetermined significance ASC‐US; HPV, human papillomavirus; IQR, interquartile range.

### 3.2 p16/Ki67 dual stain by cytology and histology

Of the 93 included women, 66 (71.0%) had normal cytology, 8 (8.6%) had low‐grade cytology (ASC‐US and LSIL) and 19 (20.4%) had high‐grade cytology (ASC‐H and HSIL) (Table S2). DS positivity increased with the severity of cytology from normal to high‐grade (39.4% versus 94.7%, *p* < 0.001), respectively (Table S2).

Restricting the analyses to women who have undergone HPV‐based screening showed similar results to those stated above (Table S2).

### 3.3 Performance of p16/Ki67 dual‐stain cytology and cytology for CIN2+ detection

A total of 52 women (55.9%) were DS positive, of whom 29 (55.8%) had CIN2+ detected (Table 2). With respect to cytology, 27 (29.0%) women had ASC‐US+, of whom 21 (77.8%) had CIN2+ detected (Table 2). Compared with cytology, DS was more sensitive in detecting CIN2+ (70% versus 96.7%, respectively, *p* = 0.021), but less specific (90.5% versus 63.5%, *p*  < 0.001) (Table 2). The NPV of DS was significantly higher compared with cytology (97.6% versus 86.4%, *p* = 0.018), whereas the PPV of DS was significantly lower compared with the PPV for cytology (55.8% versus 77.8%, *p* = 0.001) (Table 2). When restricting to women referred as the result of HPV‐based screening (*n* = 80), we found no major differences in sensitivity, specificity, PPV and NPV compared with the overall results (Table 2).

**TABLE 2:** Sensitivity, specificity, PPV, and NPV for p16/Ki67 dual stain and cytology for CIN2+ detection in women referred for colposcopy without a visible TZ

Statistical measures	DS positive (1+) % (95% CI)	Cytology (ASC‐US+) % (95% CI)	*p* ^a^ value
All women (*n* = 93)			
Positivity	55.9 (45.6–65.8)	29.0 (20.6–39.2)	<0.001
Sensitivity	96.7 (82.8–99.9)	70.0 (50.6–85.3)	0.021
Specificity	63.5 (50.4–75.3)	90.5 (80.4–96.4)	<0.001
PPV	55.8 (41.3–69.5)	77.8 (57.7–91.4)	0.001
NPV	97.6 (87.1–99.9)	86.4 (75.7–93.6)	0.018
Women referred based on primary HPV screening (*n* = 80)			
Positivity	52.5 (41.4–63.3)	23.8 (15.6–34.4)	<0.001
Sensitivity	95.7 (78.1–99.9)	60.9 (38.5–80.3)	0.021
Specificity	64.9 (51.1–77.1)	91.2 (80.7–97.1)	<0.001
PPV	52.4 (36.4–68.0)	73.7 (48.8–90.9)	0.014
NPV	97.4 (86.2–99.9)	85.2 (73.8–93.0)	0.017

*Note*: HPV any type: HPV 16/18/other high‐risk HPV types. p16/Ki67 dual‐stain cytology threshold is one positive cell.

Abbreviations: ASC‐US+, atypical squamous cells of undetermined significance; CI, confidence interval; DS, dual stain; CIN2+, cervical intraepithelial lesion grade two or worse; NPV, negative predictive value; PPV, positive predictive value; TZ, transformation zone.

^a^ Value of *p* between cytology and DS.

Excluding unclassifiable CIN from analysis resulted in a significant decrease in PPV for DS (55.8% versus 34.1%, *p* = 0.004) and cytology (77.8 versus 52.6%, *p* = 0.004), respectively. The sensitivity, specificity and NPV for DS and cytology did not change (data not shown).

### 3.4 Performance of p16/Ki67 dual stain at different thresholds

We evaluated the performance of DS for CIN2+ detection at thresholds of one, two or more, six ore more, and more than 50 DS‐positive cells (Table 3). Increasing the threshold from one DS‐positive cell to two or more DS‐positive cells lead to a slight reduction in DS positivity from 55.9% to 51.6% (*p* = 0.56) as well as increased specificity (63.5–69.8%, *p* = 0.13) and PPV (55.8–60.4%, *p* = 0.04). Sensitivity and NPV of two or more DS‐positive cells for CIN2+ detection were almost identical compared with one‐cell cutoff (Table 3). Cutoff values at six or more and more than 50 DS‐positive cells resulted in higher specificity and PPV but at the cost of lower sensitivity and NPV compared with a cutoff of one cell (Table 3).

**TABLE 3:** Performance of p16/Ki67 dual stain for CIN2+ detection at different thresholds of p16/Ki67 dual stain positive cells (*n* = 93)

Characteristic	Positivity % (95% CI)	*p* ^a^	*p* ^b^	Sensitivity % (95% CI)	*p* ^a^	*p* ^b^	Specificity % (95% CI)	*p* ^a^	*p* ^b^	PPV % (95% CI)	*p* ^a^	*p* ^b^	NPV % (95% CI)	*p* ^a^	*p* ^b^	Youden's index
Cytology, ASC‐US+	29.0 (20.6–39.2)	Ref	NA	70.0 (50.6–85.3)	Ref	NA	90.5 (80.4–96.4)	Ref	NA	77.8 (57.7–91.4)	Ref	NA	86.4 (75.7–93.6)	Ref	NA	0.605
DS ≥1 positive cells (*n* = 52)	55.9 (45.6–65.8)	<0.001	Ref	96.7 (82.8–99.9)	0.021	Ref	63.5 (50.4–75.3)	<0.001	Ref	55.8 (41.3–69.5)	0.001	Ref	97.6 (87.1–99.9)	0.018	Ref	0.602
DS ≥2 positive cells (*n* = 48)	51.6 (41.0–62.1)	<0.002	0.557	96.7 (90.2–100.0)	0.021	1.000	69.8 (58.5–81.2)	<0.001	0.125	60.4 (46.6–74.3)	0.009	0.043	97.8 (93.5–100.0)	0.014	0.362	0.665
DS ≥6 positive cells (*n* = 38)	40.9 (30.8–51.5)	0.089	0.041	90.0 (79.3–100.0)	0.109	0.500	82.5 (73.2–91.9)	0.063	<0.001	71.1 (56.6–85.5)	0.218	0.001	94.5 (88.5–100.0)	0.071	0.238	0.725
DS > 50 positive cells (*n* = 27)	29.0 (20.1–39.4)	1.000	<0.001	76.7 (61.5–91.8)	0.774	0.031	93.7 (87.6–99.7)	0.688	<0.001	85.2 (71.8–98.6)	0.340	0.001	89.4 (82.0–96.0)	0.513	0.023	0.703

*Note*: Cytology positivity = ASC‐US+ (atypical squamous cells of undetermined significance) is defined as: ASC‐US. Youden's index = (Sensitivity + Specificity − 1).

Abbreviations: ASC‐US+, atypical squamous cells of undetermined significance; CIN2+, cervical intraepithelial neoplasia (grade 2 or higher); DS, dual stain p16/Ki67; NA, not applicable.

^a^ *p* value: comparison with cytology as reference.

^b^ *p* value: comparison with DS ≥1 as reference.

## 4 DISCUSSION

### 4.1 Main findings

Compared with cytology, DS had superior sensitivity and NPV for CIN2+ detection in women aged 45 years or above with a TZ3 at colposcopy. In contrast, specificity and PPV were significantly lower for DS compared with cytology. Using two or more DS positive cells as threshold increased the specificity and PPV without lowering sensitivity and NPV for CIN2+ detection. Taken together, these results suggest that DS‐negative women with a TZ3 can undergo follow up with repeated cervical sampling instead of diagnostic LLETZ.

### 4.2 Strengths and limitations

A key strength of this study is that all the included women had a LLETZ performed, which minimised the risk of underestimating CIN2+, as we in a recent study found that biopsies missed 54% of the CIN2+ cases detected in the LLETZ specimens.^4^ Moreover, all samples (i.e. cervical cytology, biopsies and LLETZ) were collected on the same day and analysed in the same laboratory, limiting temporal and inter‐laboratory variation, respectively. To reduce interpretation bias, cyto‐technicians were blinded to all study data except the age of the women. Limitations include the relatively small sample size making the results less robust. Moreover, we cannot rule out that excluded women differed from those included with respect to basic characteristics. However, we do not believe that this has caused selection problems, because women were excluded before the results of cytology, DS and LLETZ.

### 4.3 Interpretation

Until now, studies have mainly investigated the performance of DS for triage of HPV‐positive women in younger screening populations^8^, ^9^, ^27^ and in screen‐positive women with ASC‐US or LSIL.^28^ Fewer studies have investigated the use of DS in a referral population like ours (i.e. a high‐risk population).^29^, ^30^ In the present study, we found a significantly higher sensitivity (96.7%) and NPV (97.6%) of DS for CIN2+ detection compared with cytology, which is comparable to the above‐mentioned studies among women referred for colposcopy.^29^, ^30^ Packet et al. reported a sensitivity and NPV for DS of 95% and 94%, respectively, in a subgroup of women with inconclusive colposcopy (due to bleeding, inflammation or incomplete visualisation of the TZ).^29^ A recent study investigated the use of DS in HPV‐positive women with low‐grade cytology and TZ3 undergoing LLETZ and reported a sensitivity of 100% and NPV of 100% of DS for CIN2+ detection, whereas the specificity and PPV were 73.8% and 76.1%, respectively.^31^ These findings are in line with our results although there are important differences in the median age (68 years in our study versus 39 years in the study by Manley et al.).^31^ To our knowledge, only one study has reported the performance of DS in postmenopausal women.^32^ In that study ‘histologic’ outcome was based on biopsies from the ectocervix or cervical cytology in the case of a lack of referral for colposcopy, which may have affected the CIN2+ detection rate. They reported sensitivity, specificity, NPV and PPV of 57.1%, 94.3%, 96.3% and 46.2%, respectively, with the sensitivity being somewhat lower than in our study.^32^ This discrepancy may be explained by differences in the study design and characteristics of the study population across studies. In a recent study,^4^ we found that biopsies missed more than half of the CIN2+ cases detected in the LLETZ specimens, suggesting that the histological outcome of biopsies may be suboptimal for use as reference in women with a TZ3.

Several countries have either switched to or are currently transitioning to HPV‐based screening, with triage being performed to improve specificity and reduce the risk of unnecessary colposcopy. The most used triage strategies include cytology and partial, or in a few settings, extended HPV genotyping. However, the sensitivity of cytology decreases with increasing age and may not be the optimal triage choice for older HPV‐screen‐positive women.^33^ Other settings may use genotyping with HPV 16‐ and/or HPV 18‐positive women being referred directly for colposcopy. Given that previous studies^34^, ^35^ have reported a significant decline in the prevalence of HPV 16 and HPV 18 in cervical cancer cases with increasing age, this triage strategy may be suboptimal as well. More studies are needed to determine the best method for triage of older HPV‐screen‐positive women.

Across countries, the diagnostic workup in postmenopausal women with a TZ3 poses a major clinical challenge. There are different ways to obtain histological material from the cervix, for example, by endocervical curettage (ECC).^6^, ^36^ However, ECC has been reported to be painful and the diagnostic value of ECC to detect CIN2+ in women who have a TZ3 is not well addressed.^37^, ^38^ A recent study^31^ found DS of cervical cytology samples from HPV‐positive women with low‐grade cytology to have superior sensitivity, NPV and PPV for CIN2+ detection compared with ECC. However, as the mean age was 39 years, the value of ECC may not be fully comparable to an older postmenopausal population.^31^ Another practice in the diagnostic work‐up in postmenopausal women referred because of abnormal screening results and who have a TZ3 is a diagnostic LLETZ.^5^, ^6^, ^7^ However, a diagnostic LLETZ in all women referred for colposcopy will probably lead to a significant risk of overtreatment. For example, in a previous study we found that 68% of women referred for colposcopy had <CIN2 (normal and CIN1) detected in the LLETZ specimens.^4^

In the present study, our results suggest that DS is of great value in clinical management because of a high sensitivity and because a negative DS test provides greater reassurance against CIN2+ than a negative cytology. Hence, DS may enable a risk stratification of women into those in need of immediate excisional treatment while allowing those with a negative DS test to undergo follow up. However, future studies are needed to determine the appropriate follow‐up interval and to replicate our findings focusing on women with a TZ3.

## 5 CONCLUSION

The high NPV of DS suggests that postmenopausal women with a negative DS can safely undergo follow up with repeated cervical sampling instead of diagnostic LLETZ. Therefore, DS may be a valuable risk marker to guide clinical management of postmenopausal women with a TZ3 at colposcopy.

## 6 AUTHOR CONTRIBUTIONS

The idea and the overall design of this study were conceived by LWG, MT, BA, PB, LKP, NW, MC and AH. Recruitment, data collection, data management and analysis of samples were by RB, PNC, PB, MT and LWG. Data analyses were conducted by MT, NW, MC, AH and LWG. LWG prepared the first draft with guidance/supervision from MT, NW, MC, PB, BA, LKP, PB and AH. Critical revision of the manuscript for important intellectual content was provided by NW, MC, MT, LWG, BA, RB, PNC, PB, LKP and AH. All authors approved the final manuscript before publication.

## 8 CONFLICT OF INTERESTs

LWG, MT and LKP have received speaker's fees from Astra Zeneca, outside the submitted work. LWG, MT and BA are participating in other studies with HPV test kits and CINtec plus kits sponsored by Roche, outside the submitted work. MT has received honoraria from Roche Diagnostics for lectures on CINtec plus, outside the submitted work. AH has received reagents from Roche, Denmark for free, outside the submitted work. MC: Enduring Guidelines for Cervical Cancer Screening and Management (U.S. National Cancer Institute; Steering Committee). International Neoplasia Society's Task Force for the development of anal cancer screening guidelines (Working Group Co‐Chair). LKP: Speakers fee from Merck on subjects outside this study. PNC, RB, NW and PB report no conflict of interest. Completed disclosure of interest forms are available to view online as supporting information.

9

## 10 ETHICS STATEMENT

The project was listed at the record of processing activities for research projects in the Central Denmark Region (j.no. 1‐16‐02‐528‐18) and was deemed exempt from the need for ethics approval (j.no:1‐10‐72‐4‐17).

## Supporting information


Table S1

Table S2

ICMJE

ICMJE

ICMJE

ICMJE

ICMJE

ICMJE

ICMJE

ICMJE

ICMJE

ICMJE

## Data Availability

The data set generated and analysed in this study is not available for the public due to Danish legislation. Data can be made available on request from researchers who meet the criteria for access to patient's confidential data and upon approval from the Danish Data Protection Agency.

## References

[1] Landy R , Pesola F , Castanon A , Sasieni P . Impact of cervical screening on cervical cancer mortality: estimation using stage‐specific results from a nested case‐control study. Br J Cancer. 2016;115(9):1140–6. 10.1038/bjc.2016.290 27632376 PMC5117785

[2] Wentzensen N , Walker JL , Gold MA , Smith KM , Zuna RE , Mathews C , et al. Multiple biopsies and detection of cervical cancer precursors at colposcopy. J Clin Oncol. 2015;33(1):83–9. 10.1200/JCO.2014.55.9948 25422481 PMC4268255

[3] Prendiville W , Sankaranarayanan R . Colposcopy and treatment of cervical precancer. Lyon: International Agency for Research on Cancer; 2017 [cited 2022 Mar 1]. Available from:. https://publications.iarc.fr/555 33689255

[4] Gustafson LW , Hammer A , Bennetsen MH , Kristensen CB , Majeed H , Petersen LK , et al. Cervical intraepithelial neoplasia in women with transformation zone type 3: cervical biopsy versus large loop excision. BJOG. 2022:1–9. 10.1111/1471-0528.17200 PMC9796102 35488417

[5] Gynecology SSoOa . SFOG‐riktlinje av NVP för cervixcancerprevention. Updated 190330 [cited 2020 Oct 30]. Available from: https://www.sfog.se/media/336482/sfog‐riktlinje‐190330_revhs‐190413.pdf

[6] Frazer I. Guidelines for the management of screen‐detected abnormalities, screening in specific populations and investigation of abnormal vaginal bleeding. Cancer Council Australia Cervical Cancer Screening Guidelines Working Party. National Cervical Screening Program Sydney: Cancer Council Australia [cited 2020 Oct 30]. Available from: https://wiki.cancer.org.au/australia/Guidelines:Cervical_cancer/Screening

[7] Petersen L , Schroll J , Dolleris BB , Bundgaard A , Kirschner B , Booth BB , et al National clinical guideline for cervical dysplasia. Examination, treatment, and management of women aged 60 and older [Only in Danish]. DSOG [cited 2022 Jan 6]. Available from: https://www.sst.dk/‐/media/Opgaver/Patientforl%C3%B8b‐og‐kvalitet/NKR/Puljefinansierede‐NKR/pdf‐version‐af‐ublished_guideline_2633.ashx?la=da&hash=0809DD9A773B341B3D08AE73C9361E2AB84029E8

[8] Wentzensen N , Clarke MA , Bremer R , Poitras N , Tokugawa D , Goldhoff PE , et al. Clinical evaluation of human papillomavirus screening with p16/Ki‐67 dual stain triage in a large organized cervical cancer screening program. JAMA Intern Med. 2019;179(7):881–8. 10.1001/jamainternmed.2019.0306 31081870 PMC6515572

[9] Wentzensen N , Fetterman B , Castle PE , Schiffman M , Wood SN , Stiemerling E , et al. p16/Ki‐67 dual stain cytology for detection of cervical precancer in HPV‐positive women. J Natl Cancer Inst. 2015;107(12):djv257. 10.1093/jnci/djv389 26376685 PMC4675094

[10] Wright TC Jr , Stoler MH , Ranger‐Moore J , Fang Q , Volkir P , Safaeian M , et al. Clinical validation of p16/Ki‐67 dual‐stained cytology triage of HPV‐positive women: results from the IMPACT trial. Int J Cancer. 2021;150:461–71. 10.1002/ijc.33812 34536311 PMC9293341

[11] Gustinucci D , Benevolo M , Cesarini E , Mancuso P , Passamonti B , Giaimo MD , et al. Accuracy of different triage strategies for human papillomavirus positivity in an Italian screening population. Int J Cancer. 2022;150(6):952–60. 10.1002/ijc.33858 34706093

[12] Tranberg M , Petersen LK , Elfström KM , Hammer A , Blaakær J , Bennetsen MH , et al. Expanding the upper age limit for cervical cancer screening: a protocol for a nationwide non‐randomised intervention study. BMJ Open. 2020;10(11):e039636. 10.1136/bmjopen-2020-039636 PMC7646343 33154056

[13] NSLS . The National Steering Committee for Cervical Cancer Screening [cited 2020 Sep 9]. Available from: https://www.regioner.dk/nsls/viden

[14] Petersen L . Cervix dysplasia – guideline. Danish Society of Obstetrics and Gynecology [cited 2020 Feb 2]. Available from: https://www.dsog.dk/gynkologi

[15] Colposcopy TIFoCPa . IFCPC nomenclature [cited Jul 2021]. Available from: https://ifcpc.org/medical‐professionals/ifcpc‐nomenclature/

[16] Quaas J , Reich O , Frey Tirri B , Küppers V . Explanation and use of the colposcopy terminology of the IFCPC (International Federation for Cervical Pathology and Colposcopy) Rio 2011. Geburtshilfe Frauenheilkd. 2013;73(9):904–7. 24771940 10.1055/s-0033-1350824 PMC3859163

[17] Erichsen R , Lash TL , Hamilton‐Dutoit SJ , Bjerregaard B , Vyberg M , Pedersen L . Existing data sources for clinical epidemiology: the Danish National Pathology Registry and Data Bank. Clin Epidemiol. 2010;2:51–6. 20865103 10.2147/clep.s9908 PMC2943174

[18] Schmidt M , Pedersen L , Sorensen HT . The Danish Civil Registration System as a tool in epidemiology. Eur J Epidemiol. 2014;29(8):541–9. 10.1007/s10654-014-9930-3 24965263

[19] Nayar RWD . The Bethesda system for reporting cervical cytology. definitions, criteria, and explanatory notes. 3rd ed. Berlin: Springer International Publishing; 2015.

[20] Heideman DA , Hesselink AT , Berkhof J , van Kemenade F , Melchers WJ , Daalmeijer NF , et al. Clinical validation of the cobas 4800 HPV test for cervical screening purposes. J Clin Microbiol. 2011;49(11):3983–5. 21880968 10.1128/JCM.05552-11 PMC3209101

[21] Hammer A , Gustafson LW , Christensen PN , Brøndum R , Andersen B , Andersen RH , et al. Implementation of p16/Ki67 dual stain cytology in a Danish routine screening laboratory: importance of adequate training and experience. Cancer Med. 2020;9:8235–42. 10.1002/cam4.3399 32894896 PMC7643653

[22] Roche . Roche receives FDA approval for CINtec PLUS cytology test to aid clinicians in improving cervical cancer prevention. Roche [cited 2021 Jan 6]. Available from: https://www.roche.com/media/releases/med‐cor‐2020‐03‐11.htm

[23] Richart RM . Natural history of cervical intraepithelial neoplasia. Clin Obstet Gynecol. 1967;10(4):748–84.4172733

[24] Harris PA , Taylor R , Minor BL , Fernandez M , O’Neal L , McLeod L , et al. The REDCap consortium: building an international community of software platform partners. J Biomed Inform. 2019;95:103208. 31078660 10.1016/j.jbi.2019.103208 PMC7254481

[25] Harris PA , Taylor R , Thielke R , Payne J , Gonzalez N , Conde JG . Research electronic data capture (REDCap) – a metadata‐driven methodology and workflow process for providing translational research informatics support. J Biomed Inform. 2009;42(2):377–81. 10.1016/j.jbi.2008.08.010 18929686 PMC2700030

[26] Leisenring W , Alonzo T , Pepe MS . Comparisons of predictive values of binary medical diagnostic tests for paired designs. Biometrics. 2000;56(2):345–51. 10.1111/j.0006-341x.2000.00345.x 10877288

[27] Uijterwaal MH , Polman NJ , Witte BI , van Kemenade FJ , Rijkaart D , Berkhof J , et al. Triaging HPV‐positive women with normal cytology by p16/Ki‐67 dual‐stained cytology testing: baseline and longitudinal data. Int J Cancer. 2015;136(10):2361–8. 10.1002/ijc.29290 25345358

[28] Bergeron C , Ikenberg H , Sideri M , Denton K , Bogers J , Schmidt D , et al. Prospective evaluation of p16/Ki‐67 dual‐stained cytology for managing women with abnormal Papanicolaou cytology: PALMS study results. Cancer Cytopathol. 2015;123(6):373–81. 10.1002/cncy.21542 25891096

[29] Packet B , Poppe W , Weynand B , Vanherck M . The use of p16/Ki‐67 dual staining technology on cervical cytology of patients undergoing a LLETZ procedure. Eur J Obstet Gynecol Reprod Biol. 2018;228:191–6. 30007246 10.1016/j.ejogrb.2018.06.025

[30] Wentzensen N , Schwartz L , Zuna RE , Smith K , Mathews C , Gold MA , et al. Performance of p16/Ki‐67 immunostaining to detect cervical cancer precursors in a colposcopy referral population. Clin Cancer Res. 2012;18(15):4154–62. 10.1158/1078-0432.CCR-12-0270 22675168 PMC4237612

[31] Manley K , Patel A , Pawade J , Glew S , Hunt K , Villeneuve N , et al. The use of biomarkers and HPV genotyping to improve diagnostic accuracy in women with a transformation zone type 3. Br J Cancer. 2022;126(1):91–9. 10.1038/s41416-021-01539-y 34716397 PMC8727619

[32] Dovnik A , Repse FA . P16/Ki‐67 immunostaining in the triage of postmenopausal women with low‐grade cytology results. J Low Genit Tract Dis. 2020;24(3):235–7. 10.1097/LGT.0000000000000539 32574476

[33] Gyllensten U , Lindell M , Gustafsson I , Wilander E . HPV test shows low sensitivity of Pap screen in older women. Lancet Oncol. 2010;11(6):509–10. 10.1016/S1470-2045(10)70064-4 20522375

[34] Hammer A , Mejlgaard E , Gravitt P , Høgdall E , Christiansen P , Steiniche T , et al. HPV genotype distribution in older Danish women undergoing surgery due to cervical cancer. Acta Obstet Gynecol Scand. 2015;94(11):1262–8. 10.1111/aogs.12731 26300424

[35] Hammer A , Rositch A , Qeadan F , Gravitt PE , Blaakaer J . Age‐specific prevalence of HPV16/18 genotypes in cervical cancer: a systematic review and meta‐analysis. Int J Cancer. 2016;138(12):2795–803. 10.1002/ijc.29959 26661889

[36] Perkins RB , Guido RS , Castle PE , Chelmow D , Einstein MH , Garcia F , et al. 2019 ASCCP risk‐based management consensus guidelines for abnormal cervical cancer screening tests and cancer precursors. J Low Genit Tract Dis. 2020;24(2):102–31. 10.1097/lgt.0000000000000525 32243307 PMC7147428

[37] Gage JC , Duggan MA , Nation JG , Gao S , Castle PE . Detection of cervical cancer and its precursors by endocervical curettage in 13,115 colposcopically guided biopsy exams. Am J Obstet Gynecol. 2010;203(5):481.e1–9. 10.1016/j.ajog.2010.06.048 PMC2975767 20800216

[38] Driggers RW , Zahn CM . To ECC or not to ECC: the question remains. Obstet Gynecol Clin North Am. 2008;35(4):583–97; viii. 10.1016/j.ogc.2008.09.007 19061818

