# Supplementary material for: Clinical utility of p16/Ki67 dual‐stain cytology for detection of cervical intraepithelial neoplasia grade two or worse in women with a transformation zone type 3: A cross‐sectional study
Source: BJOG. 2022 Jun 22;130(2):202–9. doi: 10.1111/1471-0528.17248 (PMC10084097; doi:10.1111/1471-0528.17248)
Supplement: Supplementary file 1 — Table S1 [file BJO-130-202-s008.docx]

**Supporting Tables:**

**Table S1:** Management of screen-positive women according to the Danish cervical cancer screening guideline.

|  | **Result of primary screening method** | **Reflex testing** | **Management** |
| --- | --- | --- | --- |
| **Women aged 23-59 who undergo cytology-based screening** | | | |
|  | ASC-US in women < 30 yrs. | Reflex HPV testing not required | Repeat cytology test at 6 months |
|  | ASC-US in women ≥30 | Positive reflex HPV test | Direct referral to colposcopy |
|  |  | Negative reflex HPV test | Return to screening program |
|  | LSIL | Reflex HPV testing not recommended | Repeat cytology test after 6 months |
|  | ASC-H, AGC, AIS, or HSIL | Reflex HPV testing not recommended | Direct referral to colposcopy |
| **Women aged 30-59 who undergo HPV-based screening** | | | |
|  | HPV positive | ASC-US, LSIL on cytology triage^a^ | Additional triage dependent on region |
|  |  | AGC, ASC-H, HSIL, AIS on cytology triage^a^ | Direct referral to colposcopy |
|  |  | Normal cytology on cytology triage | Repeat HPV testing after one year |
| **Women aged 60-64 who undergo HPV-based screening** | | | |
|  | Positive for HPV 16 or 18^b^ | Not required | Direct referral to colposcopy |
|  | Positive for other HPV types | ASC-US or worse on reflex cytology | Direct referral to colposcopy |
|  |  | Normal on reflex cytology | Repeat HPV test after one year^c^ |

Abbreviations: ASC-US: atypical squamous cells of undetermined significance, LSIL: low-grade squamous intraepithelial lesion, HSIL: High-grade squamous intraepithelial lesion, Atypical squamous cells-cannot exclude HSIL (ASC-H), Atypical glandular cells (AGC), Adenocarcinoma in situ (AIS), HPV: human papilloma virus, hrHPV: high-risk human papilloma virus. ^a^ Further triage will be conducted if cytology triage shows ASC-US or LSIL, ^b^ Including high risk HPV other types in combination with 16 and/ or 18. ^c^ Two positive tests with persistent HPV high risk other types of infection will lead to referral to colposcopy.
