# Supplementary material for: Clinical utility of p16/Ki67 dual‐stain cytology for detection of cervical intraepithelial neoplasia grade two or worse in women with a transformation zone type 3: A cross‐sectional study
Source: BJOG. 2022 Jun 22;130(2):202–9. doi: 10.1111/1471-0528.17248 (PMC10084097; doi:10.1111/1471-0528.17248)
Supplement: Supplementary file 2 — Table S2 [file BJO-130-202-s004.docx]

**Supporting Tables:**

**Table S2:** p16/Ki67 dual stain positivity by cytology and histology (LLETZ) result

| **Cytology result** | **Histology result (LLETZ) (%)** | | |
| --- | --- | --- | --- |
|  | **Total** | **<CIN2** | **CIN2+** |
| **All included women (n=93)** | | | |
| **Normal cytology (n)**  DS+ | 66  26 (39.4) | 57  17 (29.8) | 9  9 (100.0) |
| **Low-grade cytology (n)**  DS+ | 8  8 (100.0) | 3  3 (100.0) | 5  5 (100.0) |
| **High-grade cytology (n)**  DS+ | 19  18 (94.7) | 3  3 (100.0) | 16  15 (93.8) |
| **Total**  DS+ | 93 (100.0)  52 (55.9) | 63 (100.0)  23 (36.5) | 30 (100.0)  29 (96.7) |
| **Women referred based on primary HPV screening (n = 80)** | | | |
| **Normal cytology (n)**  DS+ | 61  24 (39.3) | 52  15 (28.8) | 9  9 (100.0) |
| **Low-grade cytology (n)**  DS+ | 5  5 (100.0) | 2  2 (100.0) | 3  3 (100.0) |
| **High-grade cytology (n)**  DS+ | 14  13 (92.9) | 3  3 (100.0) | 11  10 (90.9) |
| **Total**  DS+ | 80 (100.0)  42 (52.5) | 57 (100.0)  20 (35.1) | 23 (100.0)  22 (95.7) |

Abbreviations: Dual stain positivity in % was calculated within each cytology group. Low grade cytology was defined as: Atypical squamous cells of undetermined significance (ASC-US) and low-grade squamous intraepithelial lesions (LSIL). High grade cytology was defined as: Atypical squamous cells, cannot rule out high-grade squamous intraepithelial lesion (ASC-H) and high-grade squamous intraepithelial lesion (HSIL).
