# Supplementary material for: Clinical utility of p16/Ki67 dual‐stain cytology for detection of cervical intraepithelial neoplasia grade two or worse in women with a transformation zone type 3: A cross‐sectional study
Source: BJOG. 2022 Jun 22;130(2):202–9. doi: 10.1111/1471-0528.17248 (PMC10084097; doi:10.1111/1471-0528.17248)
Supplement: Supplementary file 3 — ICMJE [file BJO-130-202-s010.pdf]

ICMJE DISCLOSURE FORM

Date: 5/1/2022

Your Name: Line Winther Gustafson

Manuscript Title: Clinical utility of p16/Ki67 dual stain cytology for detection of cervical intraepithelial neoplasia grade two or worse in women with a transformation zone type 3: a cross sectional study

Manuscript Number (if known): Manuscript ID BJOG-22-0373

In the interest of transparency, we ask you to disclose all relationships/activities/interests listed below that are related to the content of your manuscript. "Related" means any relation with for-profit or not-for-profit third parties whose interests may be affected by the content of the manuscript. Disclosure represents a commitment to transparency and does not necessarily indicate a bias. If you are in doubt about whether to list a relationship/activity/interest, it is preferable that you do so.

The author's relationships/activities/interests should be defined broadly. For example, if your manuscript pertains to the epidemiology of hypertension, you should declare all relationships with manufacturers of antihypertensive medication, even if that medication is not mentioned in the manuscript.

In item #1 below, report all support for the work reported in this manuscript without time limit. For all other items, the time frame for disclosure is the past 36 months.

		Name all entities with whom you have this relationship or indicate none (add rows as needed)	Specifications/Comments (e.g., if payments were made to you or to your institution)
		**Time frame: Since the initial planning of the work**	
1	All support for the present manuscript (e.g., funding, provision of study materials, medical writing, article processing charges, etc.) **No time limit for this item.**	☐ None	
		This study was supported by a grant from the Health Research Foundation of Central Denmark Region (A3234) and Dagmar Marshalls Foundation	Salery for PhD student Line Winther Gustafson.
		This study was supported by a grant from, A.P. Moller Foundation, Else and Mogens Wedell-Wedellsborgs Foundation and Fabricant Einar Willumsens Mindelegat	Running cost for Line Winther Gustafson's PhD project
			Click the tab key to add additional rows.
		**Time frame: past 36 months**	
2	Grants or contracts from any entity (if not indicated in item #1 above).	☒ **None**	
3	Royalties or licenses	☒ **None**	

		Name all entities with whom you have this relationship or indicate none (add rows as needed)	Specifications/Comments (e.g., if payments were made to you or to your institution)
4	Consulting fees	☒ None	
5	Payment or honoraria for lectures, presentations, speakers bureaus, manuscript writing or educational events	☐ None	
		Astra Zeneca	Line W. Gustafson has received speaker's fee from Astra Zeneca
6	Payment for expert testimony	☒ None	
7	Support for attending meetings and/or travel	☐ None	
		Danish Cancer Society travel grant	The purpose of the travel was to visit dr. Nicolas Wentzensen at National Cancer Institute, US.
8	Patents planned, issued or pending	☒ None	
9	Participation on a Data Safety Monitoring Board or Advisory Board	☒ None	
10	Leadership or fiduciary role in other board, society, committee or advocacy group, paid or unpaid	☒ None	

		Name all entities with whom you have this relationship or indicate none (add rows as needed)	Specifications/Comments (e.g., if payments were made to you or to your institution)
**11**	Stock or stock options	☒ **None**	
**12**	Receipt of equipment, materials, drugs, medical writing, gifts or other services	☐ **None**	
		Roche Diagnostics.	Line Winther Gustafson is participating in other studies with HPV test kits and CINtec plus kits sponsored by Roche
**13**	Other financial or non-financial interests	☒ **None**	

Please place an "X" next to the following statement to indicate your agreement:

☒ I certify that I have answered every question and have not altered the wording of any of the questions on this form.
